# Structure and Activity of a Selective Antibiofilm Peptide SK-24 Derived from the NMR Structure of Human Cathelicidin LL-37

**DOI:** 10.3390/ph14121245

**Published:** 2021-11-30

**Authors:** Yingxia Zhang, Jayaram Lakshmaiah Narayana, Qianhui Wu, Xiangli Dang, Guangshun Wang

**Affiliations:** Department of Pathology and Microbiology, College of Medicine, University of Nebraska Medical Center, 985900 Nebraska Medical Center, Omaha, NE 68198-5900, USA; yingxiazhang@hotmail.com (Y.Z.); dr.jayaramln@gmail.com (J.L.N.); 18252730036@163.com (Q.W.); xldang@ahau.edu.cn (X.D.)

**Keywords:** antimicrobial peptides, biofilms, cathelicidin, LL-37 oligomerization, SK-24, structure-based design

## Abstract

The deployment of the innate immune system in humans is essential to protect us from infection. Human cathelicidin LL-37 is a linear host defense peptide with both antimicrobial and immune modulatory properties. Despite years of studies of numerous peptides, SK-24, corresponding to the long hydrophobic domain (residues 9–32) in the anionic lipid-bound NMR structure of LL-37, has not been investigated. This study reports the structure and activity of SK-24. Interestingly, SK-24 is entirely helical (~100%) in phosphate buffer (PBS), more than LL-37 (84%), GI-20 (75%), and GF-17 (33%), while RI-10 and 17BIPHE2 are essentially randomly coiled (helix%: 7–10%). These results imply an important role for the additional N-terminal amino acids (likely E16) of SK-24 in stabilizing the helical conformation in PBS. It is proposed herein that SK-24 contains the minimal sequence for effective oligomerization of LL-37. Superior to LL-37 and RI-10, SK-24 shows an antimicrobial activity spectrum comparable to the major antimicrobial peptides GF-17 and GI-20 by targeting bacterial membranes and forming a helical conformation. Like the engineered peptide 17BIPHE2, SK-24 has a stronger antibiofilm activity than LL-37, GI-20, and GF-17. Nevertheless, SK-24 is least hemolytic at 200 µM compared with LL-37 and its other peptides investigated herein. Combined, these results enabled us to appreciate the elegance of the long amphipathic helix SK-24 nature deploys within LL-37 for human antimicrobial defense. SK-24 may be a useful template of therapeutic potential.

## 1. Introduction

In insects, different antimicrobial peptides (AMPs) are induced in response to the infection of invading pathogens. In the case of Gram-positive bacteria and fungi, drosomycin, metchnikowin, and defensin are expressed via the Toll pathway. In contrast, diptericin, drosocin, cecropins, and attacins are expressed to combat primarily Gram-negative bacteria via the Imd pathway [1]. Humans also deploy host defense peptides to fend off invading pathogens [2]. Lysozyme, isolated by Alexander Fleming in 1922, is regarded as the first antimicrobial protein [3]. It contains four pairs of disulfide bonds. Since 1985, additional disulfide-linked AMPs, such as human α- and β-defensins, have been discovered [4,5]. Subsequent bioinformatic studies predicted two dozen of human defensins with three characteristic disulfide bonds [6]. However, only one cathelicidin gene is predicted in the human genome based on a highly conserved precursor region [7]. The mature region encodes a 37-residue linear antimicrobial peptide starting with a pair of leucines, thereby dubbed LL-37. This peptide has a net charge of +6 (six lysines, five arginines, no histidine, three glutamic acids, and two aspartic acids) and a hydrophobic content of 35% based on the upgraded antimicrobial peptide database (APD; https://aps.unmc.edu (accessed on 29 September 2021)) [8]. It does not contain methionine, alanine, tryptophan, histidine, tyrosine, and cysteine. As a consequence, LL-37 is unable to form disulfide bonds. This peptide has been detected in the saliva, skin, and lung. Sunlight and vitamin D can induce the expression of LL-37 [9] to combat bacteria, fungi, viruses, and parasites. Besides, this human cathelicidin inhibits biofilm formation, suppresses the TLR-4 pathway by neutralizing bacterial lipopolysaccharides (LPS), regulates host cytokine release, and promotes wound healing [10,11,12,13,14,15].

**Figure 1:**
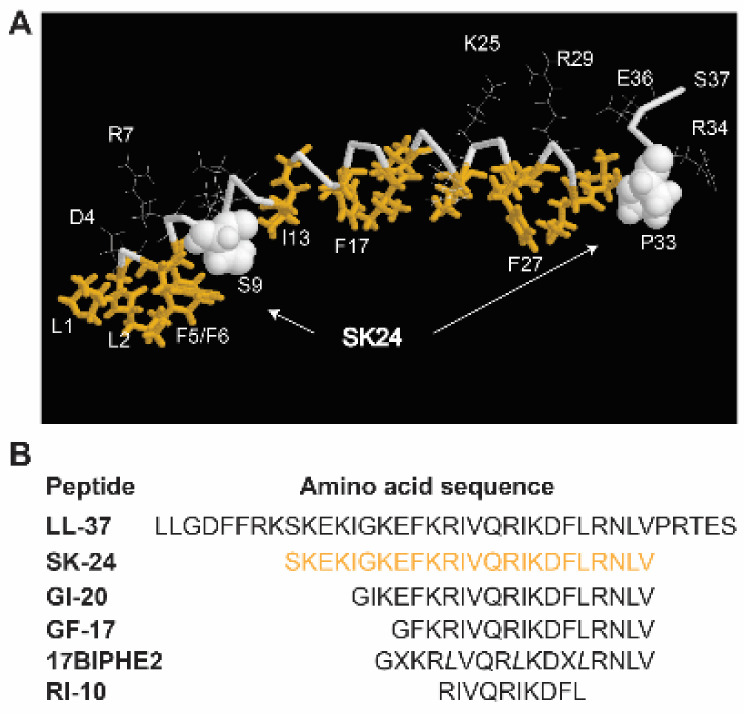
(**A**) Identification of the SK-24 peptide based on the three-dimensional structure of human cathelicidin LL-37 determined by 3D NMR spectroscopy (PDB ID: 2K6O) [26]. In the figure, hydrophobic amino acids are in gold and backbone in grey. Amino acids S9 and P33 are represented in the space-filling model. (**B**) The amino acid sequences of LL-37 and its derived peptides. The SK-24 sequence (residues 9–32) is in gold. X represents biphenylalanines, and italic *L* indicates D-leucines in 17BIPHE2. All LL-37 fragments are C-terminally amidated.

The broad-spectrum antimicrobial activity of LL-37 stimulated the interest in developing this molecule into peptide antibiotics to combat antibiotic-resistant pathogens. Numerous labs attempted to identify the active regions of LL-37 [16,17,18,19,20,21,22,23,24,25,26,27]. While the N-terminal region of LL-37 is less active [24], multiple antimicrobial peptides are discovered from the central region of LL-37 [14,15]. Braff et al. identified multiple active peptides via a library approach [16]. A C-terminal LL-37 fragment was discovered via proteolysis [17]. We identified the major antimicrobial and anticancer region GF-17 corresponding to residues 17–32 of LL-37 via the NMR-trim technology [18]. Another peptide, GI-20, derived from residues 13–32, was found to have antiviral, antibacterial, and spermicidal activities [28,29,30,31]. Based on the 3D-NMR-determined LL-37 structure bound to membrane mimetic micelles [26], it became clear to us that a long helical region starting at residue S9 and ending before P33 of LL-37 has not been studied (Figure 1). This study investigates the structure and activity of this long helical region. A peptide corresponding to residues 9–32 of LL-37 was synthesized and named SK-24 according to the LL-37 nomenclature. In this convention, SK represents the first two amino acids of the new peptide, and 24 indicates the length of the peptide. Additionally, we refer to amino acids in SK-24 based on their positions in the parent peptide, LL-37. Thus, SK corresponds to residues 9 and 10 of LL-37, respectively. For comparison, we also included shorter LL-37 peptides (GI-20, GF-17, 17BIPHE2, and RI-10) we obtained previously [18,26,30,32]. Our study shines light on the region of LL-37 involved in oligomerization. Remarkably, SK-24 shows poor cytotoxicity to human erythrocytes but potency against bacteria in planktonic and biofilm forms. Such a combined feature for a peptide is desired in search for novel antimicrobials to combat antibiotic-resistant pathogens.

## 2. Results

### 2.1. Peptide Design and Properties

In the membrane-bound LL-37 structure determined by 3D NMR spectroscopy, a hydrophobic gap exists (Figure 1A). The location of a hydrophilic S9 on the hydrophobic surface of LL-37 splits the long helix into two hydrophobic domains (gold sticks). The SK-24 peptide we identified here corresponds to the long hydrophobic domain [26]. The amino acid sequence of SK-24, along with LL-37 and its derived antimicrobial peptides, is depicted in Figure 1B. Peptide parameters, including net charge, hydrophobic content, Boman index, GRAVY, hydrophobicity, and hydrophobic moment of each peptide, were calculated using online tools, such as the APD [8] and HeliQuest [33] (Table 1). The HPLC retention time [34] measured for each peptide is also provided in Table 1. Except for the shortest RI-10 (net charge +3), LL-37 and its other derived peptides have net charges +5 or +6, above the average net charge (+3.3) of natural antimicrobial peptides registered in the APD [8,35,36]. Overall, the hydrophobic percentages (Pho) of these peptides calculated in the APD are proportional to the hydrophobicity and hydrophobic moment calculated by HeliQuest in Table 1. Of note, LL-37 has the highest net charge (+6) and Boman index but the lowest Pho%, GRAVY, and hydrophobic moment.

### 2.2. Secondary Structure in Phosphate Buffer and Membrane Mimetic Micelles

**Figure 2:**
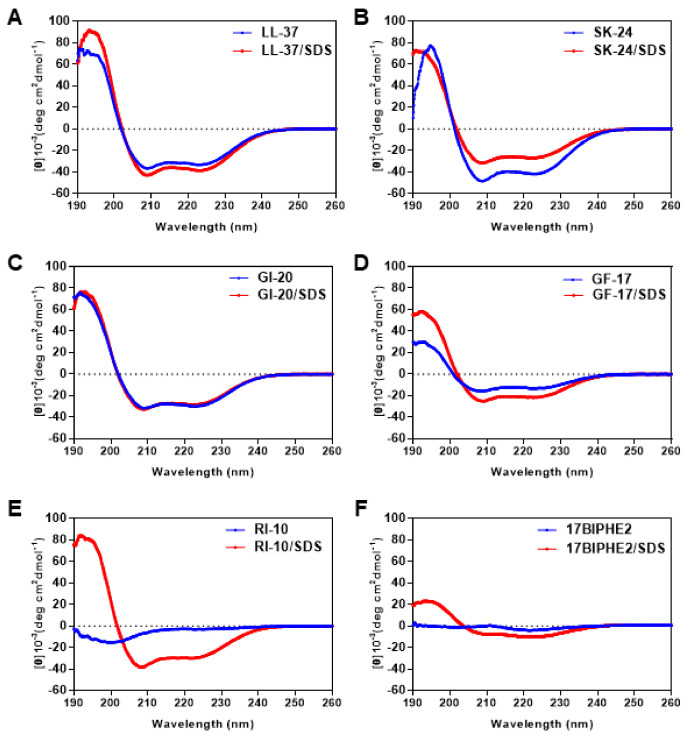
Circular dichroism spectra of SK-24 and its related peptides in 10 mM PBS buffer and in the presence of SDS micelles at pH 7 and 25 °C. The peptides include (**A**) LL-37, (**B**) SK-24, (**C**) GI-20, (**D**) GF-17, (**E**) RI-10, and (**F**) 17BIPHE2. The peptide concentration was fixed at 0.5 mM in all the spectra, and the peptide/SDS molar ratio was 1:60.

LL-37 became helical upon association with membranes or their mimics based on both circular dichroism (CD) and NMR studies [21,26,37]. To provide evidence for helix formation, we recorded CD spectra of SK-24 as well as LL-37 and other peptides (Figure 2). Because CD is especially suitable to follow the change in peptide conformation under different conditions, we acquired spectra in both phosphate buffer (PBS) and the micelles of sodium dodecyl sulfate (SDS). SDS is a widely utilized model to mimic anionic lipids of bacterial membranes. In the case of LL-37, we observed a similar ordered–disordered structural pattern in SDS, dioctanoyl phosphatidylglycerol (D8PG), and lipopolysaccharides (LPS). Such a structural pattern is in full agreement with the heteronuclear ^15^N NOE plot [26]. For LL-23, we found the same secondary structure in SDS, D8PG, and dodecylphosphocholine (DPC) [24]. Usually, a typical linear peptide does not have an ordered structure in PBS, and only becomes helical after binding to membranes. We estimated peptide helicity based on the 222 nm band. In SDS, RI-10 was much more helical (75%) than 17BIPHE2 (25%). The poor helicity of 17BIPHE2 due to the insertion of D-amino acids at three sites agrees with the 3D structure determined by NMR spectroscopy [18]. Other peptides were rather different in that they were more or less helical already in PBS at the concentration we studied (0.5 mM). In the case of LL-37 and GF-17, they became more helical in SDS micelles. Noticeably, SK-24 and GI-20, especially SK-24, were slightly less helical in SDS (Table 2). The same conclusion could be derived based on R1 and R2 values [38]. R1 is the molar ellipticity ratio at 192 and 206 nm, whereas R2 is the molar ellipticity ratio at 222 and 208 nm. In complex with SDS micelles, LL-37, SK-24, GI-20, GF-17, and RI-10 displayed similar R1 and R2 values (Table 2), indicating similar helical conformations. Only 17BIPHE2 was very different as a consequence of a distorted backbone conformation as determined by NMR [18]. In PBS, GF-17, GI-20, SK-24, and LL-37 had similar R2, although R1 varied from −1.14 to −2.48.

### 2.3. SK-24 Has a Broad Antibacterial Activity

The minimal inhibitory concentrations (MICs) of SK-24 against various clinical and antibiotic-resistant pathogens are listed in Table 3. SK-24 showed good antibacterial activity against *Enterococcus faecium*, *Staphylococcus aureus* USA300, and *Acinetobacter baumannii* with MIC values in the range of 2–8 μM. It inhibited the growth of *Escherichia coli* E423-17 at 16 μM and had poor effect on *Pseudomonas aeruginosa* E411-17 and *Klebsiella pneumoniae* E406-17 at 32 μM. For comparison, we also tested the antibacterial activity of LL-37 and its select peptides we obtained previously. The antimicrobial spectrum of SK-24 resembled GI-20 and GF-17. SK-24 was superior to LL-37, which displayed higher MIC values in most of the cases even in 15% tryptic soy broth (TSB). We recently found similar MIC values for a short lipoLL-37 peptide in 100% TSB and Mueller Hinton Broth (MHB) [39]. It appeared that 17BIPHE2, engineered based on GF-17 [32], was most potent in killing these pathogens with MIC in the range of 2–8 µM. Consistent with our previous finding [26], RI-10 did not show any inhibitory activity at 32 µM (Table 3). The loss of the antimicrobial activity of RI-10 may be explained by the shortest HPLC retention time, indicative of a least hydrophobic peptide in Table 1.

### 2.4. Antibiofilm Activity of SK-24

**Figure 3:**
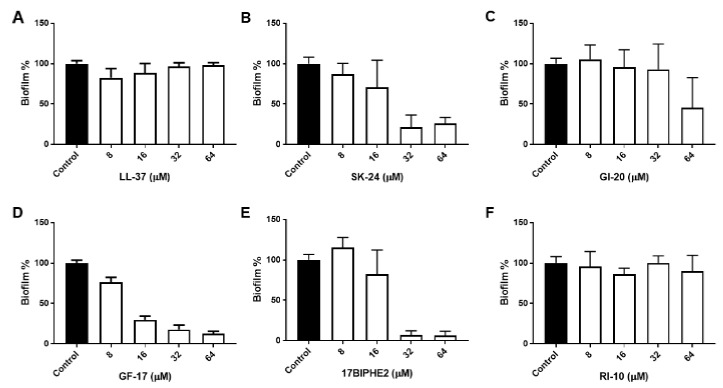
Antibiofilm activity of SK-24 and LL-37 peptides against *A. baumannii* B28-16: (**A**) LL-37, (**B**) SK-24, (**C**) GI-20, (**D**) GF-17, (**E**) 17BIPHE2, and (**F**) RI-10. Biofilms were established for 48 h.

**Figure 4:**
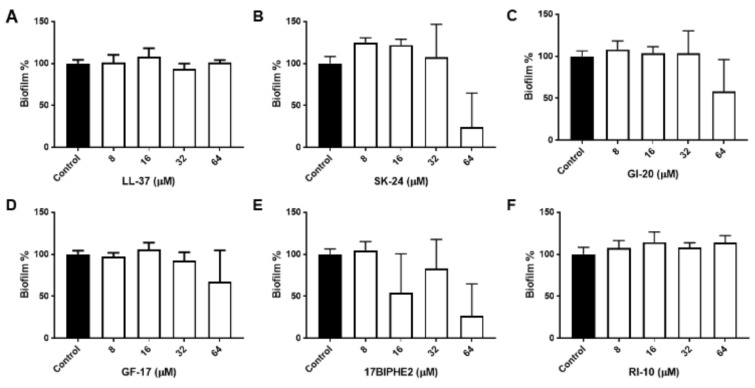
Antibiofilm activity of SK-24 and LL-37 peptides against *A. baumannii* B28-16: (**A**) LL-37, (**B**) SK-24, (**C**) GI-20, (**D**) GF-17, (**E**) 17BIPHE2, and (**F**) RI-10. Biofilms were established for 72 h.

To further gauge peptide activity, we then compared the antibiofilm activity of SK-24 in disrupting the preformed biofilms of Gram-negative *A. baumannii* B28-16 and Gram-positive *S. aureus* USA300 LAC. Again, LL-37 and its selected peptides were included for comparison. The biofilms of *A. baumannii* were formed for 24 h (Figure S1), 48 h (Figure 3), and 72 h (Figure 4), respectively. In all the cases, LL-37 and RI-10 treated at 8, 16, 32, or 64 μM were unable to disrupt the preformed biofilms of *A. baumannii*. However, SK-24, as well as GF-17, GI-20, and 17BIPHE2, all reduced biofilms established in 24 h in a dose-dependent manner (Figure S1). Here, the biofilm-elimination ability of SK-24 appeared to be comparable to that of GI-20 and GF-17 but was slightly less effective than that of 17BIPHE2. For the 48 h biofilms, we also observed dose-dependent biofilm disruption for SK-24, GF-17, and 17BIPHE2, although GI-20 became less potent (Figure 3) and only worked at 64 μM. In the case of the 72 h preformed biofilms, we only observed a clear biofilm decrease in SK-24, GF-17, GI-20, and 17BIPHE2 at 64 μM, the highest concentration tested (Figure 4). SK-24 and 17BIPHE2 appeared to be slightly more effective (~70% disruption) than GF-17 or GI-20 (30–50% disruption).

In the case of Gram-positive bacteria, we found previously that neither LL-37 nor RI-10 was able to disrupt the 24 h preformed biofilms of *S. aureus* USA300 LAC as well as other clinical strains, including *S. aureus* USA200, *S. aureus* USA400, and *S. aureus* Mu50. However, both GF-17 and 17BIPHE2 were highly effective [25]. Here, we compared the antibiofilm activity of SK-24 with that of GF-17, GI-20, and 17BIPHE2 against the 24 h preformed biofilms of *S. aureus* USA300. SK-24, similar to GF-17, GI-20, and 17BIPHE2, showed an antibiofilm effect in a dose-dependent manner (Figure S2). Only SK-24 and 17BIPHE2 reduced the *Staphylococcal* biofilms to ~25% at 32 μM.

### 2.5. SK-24 Targets Bacterial Membranes

**Figure 5:**
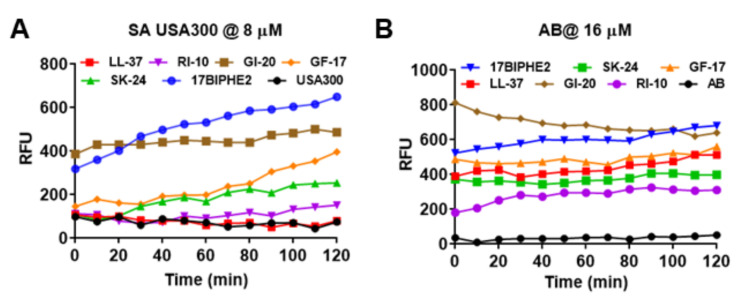
Membrane permeabilization of (**A**) *S. aureus* USA300 LAC treated at 8 µM and (**B**) *A. baumannii* treated at 16 µM of SK-24 and LL-37 peptides based on a change in the fluorescence of the dye propidium iodide.

SK-24 corresponds to the long central helix of LL-37 (Figure 1). It is reasonable to predict that the new peptide also acts on bacterial membranes like its parent peptide LL-37. To validate this, we conducted membrane permeabilization as well as depolarization experiments. Both Gram-positive *S. aureus* USA300 and Gram-negative *A. baumannii* B28-16 were utilized in the membrane permeation experiment (Figure 5), where a nonmembrane permeable dye, propidium iodide (PI), was used as an indicator. Peptides were treated at different levels, and the same trend was observed. At 8 µM, SK-24 weakly permeated the *S. aureus* membranes (Figure 5A). It was slightly better than LL-37 and RI-10 in damaging *S. aureus*. In the same experiment, however, 17BIPHE2 and GI-20 were more powerful. We also followed the membrane permeation of *A. baumannii* (Figure 5B). At 16 µM, SK-24 was similar to human LL-37 and its major antibacterial peptide GF-17. Again, GI-20 and 17BIPHE2 were superior to SK-24. Thus, there is a consensus that GI-20 and 17BIPHE2 are more potent than GF-17 and SK-24 in permeating the membranes of both Gram-positive and Gram-negative bacteria. Notably, RI-10 also showed some membrane permeation of both *S. aureus* (Figure 5A) and *A. baumannii* (Figure 5B), although it failed to inhibit the growth of these bacteria at 32 µM (Table 3).

**Figure 6:**
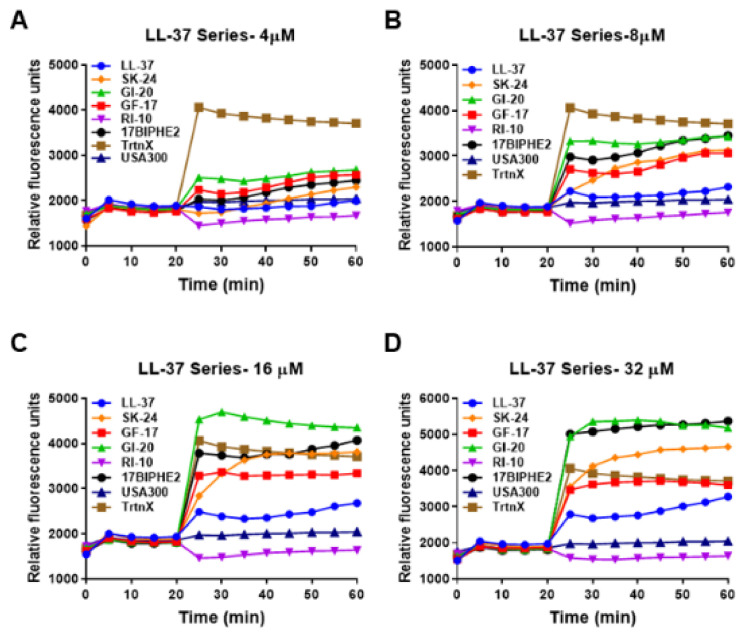
Effects of SK-24 and LL-37 peptides on membrane depolarization of the mid-log phase *S. aureus* USA300 LAC resuspended in PBS and energized with glucose for 15 min at 37 °C treated at a peptide concentration of 4 µM (**A**), 8 µM (**B**), 16 µM (**C**), and 32 µM (**D**) post-20 min dye equilibration. The detergent Triton X-100 (TrtnX, 0.1%) at a constant concentration was included as a positive control.

In the membrane depolarization experiment, SK-24 induced membrane depolarization in a dose-dependent manner (Figure 6). At 8 µM (panel B), it was stronger than LL-37 in polarizing the membranes of *S. aureus* USA300. Like membrane permeation, GI-20 and 17BIPHE2 showed a stronger membrane depolarization ability in the following order: GI-20 > 17BIPHE2 > GF-17~SK-24 > LL-37. RI-10, which is not inhibitory to bacterial growth, was unable to produce a similar effect on bacterial membranes. Similar trends were retained at higher peptide concentrations of 16 and 32 µM (panels C and D). Hence, both experiments underscored the effect of SK-24 on bacterial membranes.

### 2.6. Hemolytic Ability of SK-24

**Figure 7:**
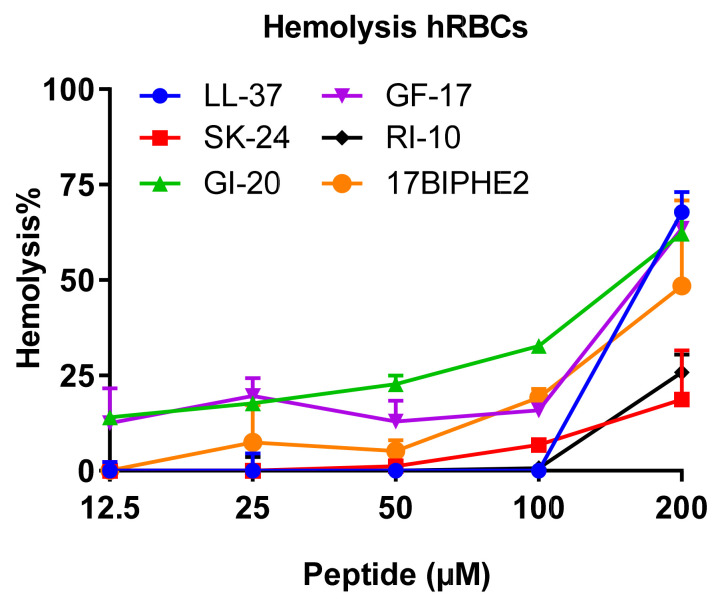
Hemolytic toxicity of SK-24 in comparison with LL-37, GF-17, GI-20, RI-10, and 17BIPHE2. The percentages of human red blood cell lysis were calculated relative to the untreated control and plotted versus the peptide concentration in the range of 12.5–200 µM.

Using the established lab protocol [40], we also compared the hemolytic activity of SK-24 with LL-37 and its peptides. RI-10 is least hemolytic, consistent with the least hydrophobicity based on the HPLC retention time (Table 1). Similar to RI-10, SK-24 displayed minimal hemolysis (<25%) even at 200 µM (Figure 7), indicating a 50% hemolytic concentration (HC_50_) greater than 200 µM. This appears to correspond to its lowest calculated hydrophobicity in Table 1. LL-37 showed no hemolysis below 100 µM. At 200 µM, however, it became rather hemolytic (70% hemolysis) with an HC_50_ of ~175 µM. Like LL-37, GF-17 showed an abrupt increase in hemolysis at 100 µM (HC_50_ ~175 µM). GI-20, with an HC_50_ of ~160 µM, appeared to be slightly more hemolytic than GF-17. As a positive control, the engineered peptide 17BIPHE2 is poorly hemolytic with an HC_50_ of ~200 µM, consistent with our previous finding, although the absolute lysis percentages differ due to a difference in blood cell batches [32].

## 3. Discussion

Human cathelicidin LL-37 is one of the best-studied antimicrobial peptides with antimicrobial and immune regulatory functions [10,11,12,13,14,15]. It is able to inhibit both Gram-positive and Gram-negative bacteria via membrane targeting. At low concentrations, especially acidic pHs, LL-37 does not have an ordered structure [11,21]. Such a randomly coiled state is frequently used as a starting point for LL-37 to attach bacterial membranes via initial electrostatic interactions where R23 is a critical cationic residue [26,41]. Subsequently, residues 2–31 of LL-37 become helical due to association with the outer-membrane LPS of Gram-negative bacteria or after binding to anionic phosphatidylglycerols (PGs) of bacteria membranes according to isotope-labeled heteronuclear NMR studies [26]. The carpet model [42] describes this state when LL-37 sits on the surface of the membranes. The toroidal pore model suggests a pore formation where both LL-37 and lipids can be located in the interface [43,44]. With an increase in peptide concentration, the peptide even lyses bacterial membranes into small pieces, forming a totally different phase of peptide–membrane complexes [45].

The membrane targeting of LL-37 can be more complex since the peptide might have already been a helix at an elevated concentration in PBS, as observed previously by others [21] and here by us (Figure 2). Size-exclusion chromatography estimated a tetramer formation. Based on NMR data, we proposed the involvement of nearly the entire LL-37 (residues 1–36) in oligomerization [11]. Here, we gained additional insight into LL-37 oligomerization by comparing a series of LL-37 fragments. Previous studies established that LL-37 became helical in PBS due to oligomerization [21]. It is interesting to note that GF-17 at 0.5 mM is less helical in PBS than either GI-20 or SK-24. It is logical to reason that those N-terminal residues absent in GF-17 are required for helix formation probably via oligomerization. Possibly, E16 is a key residue, which is absent in GF-17, but present in GI-20, SK-24, and LL-37. Interestingly, in a dimeric crystal structure of LL-37, E16 forms hydrogen bonds with S9 in the dimer interface [46], providing one possible role for this acidic residue in LL-37 oligomerization. We may apply such an interaction to the LL-37 peptides investigated here to explain the different degrees of helicity in PBS. Both S9 and E16 exist in SK-24, allowing for similar S9–E16 interactions between different chains of LL-37 in the oligomer, leading to a longer helix in PBS. Since S9 is absent in GI-20, this peptide is less helical than SK-24 in PBS. In GF-17, the lack of both S9 and E16 would explain the low helicity in PBS (Table 2).

Shai found that oligomers of LL-37 were retained in zwitterionic lipids but were dissociated into monomer in anionic lipids [22]. We found here that LL-37 became more helical in association with SDS micelles than in PBS. Likewise, GF-17 was more helical in membranes. However, GI-20 was slightly less helical in complex with SDS micelles. A less helical structure bound to SDS than in PBS is even more pronounced for SK-24 (Table 2). Such a decrease in helicity may result from electrostatic repulsions between the anionic headgroup of SDS and acidic amino acids in GI-20 (E16) and SK-24 (E11 and E16) (Figure 1). Our speculation agrees with 3D structures determined for GF-17 and GI-20 bound to SDS micelles. While GF-17 is entirely helical [47], the N-terminus of GI-20 is not. Interestingly, GI-20 is entirely helical bound to DPC or D8PG, indicating a lack of such repulsions with zwitterionic DPC or a weaker repulsion with D8PG [41,48]. Such an electrostatic repulsion might have been overcome in the case of full-length LL-37 due to the association of the N-terminal hydrophobic region (LLGDFF) of LL-37 with anionic micelles. Thus, LL-37 retains a long helix after association with either SDS or D8PG micelles [26].

Peptide length also plays an important role in determining peptide activity. The smallest antibacterial peptide KR-12 is active against *E. coli* but not *S. aureus* [26]. RI-10, obtained by further truncation of KR-12, loses its antimicrobial activity (Figure 1). FK-13 is the minimal anti-HIV peptide, indicating the significance of the N-terminal aromatic phenylalanine [30]. Further extension of FK-13 by three residues NLV at the C-terminus leads to GF-17 (G is also appended to the N-terminus to mimic some natural AMPs), which is the major antimicrobial peptide with a broad-spectrum activity against both *E. coli* and *S. aureus* [47]. During our study of the sequence–activity relationship of anti-HIV peptides, GI-20 was found to have a higher therapeutic index than GF-17 [30]. This study addresses the important question of whether SK-24, a helical peptide longer than GF-17 and GI-20 of LL-37, has any advantages in killing bacteria in the planktonic and biofilm forms. Our results indicate an antibacterial activity spectrum of SK-24 similar to GF-17 and GI-20 in the case of planktonic bacteria (Table 3), indicating that the extra residues in SK-24 (Figure 1) did not contribute substantially to MIC values. This is logical considering that the LL-37 peptides target bacterial membranes, and additional residues at the N-terminal region of SK-24 are either polar or charged. LL-37 is also reported to inhibit biofilm formation under a low peptide concentration [25,49]. However, LL-37, as well as the inactive peptide RI-10, is not effective in disrupting the preformed biofilms of MRSA, whereas the major antimicrobial peptide GF-17 and its engineered peptide 17BIPHE2 are much more effective [25]. 17BIPHE2 also inhibits *P. aeruginosa* adhesion and biofilm formation. In the more challenging case of preformed biofilms, it works even better in the presence of antibiotics [50]. In this study, the longer peptide, SK-24, is found to be more effective in disrupting preformed biofilms of *A. baumannii* and *S. aureus* than GI-20 and GF-17. Interestingly, only SK-24 shows an antibiofilm activity against the 72 h biofilm of *A. baumannii* comparable to the engineered peptide 17BIPHE2 at an elevated peptide concentration (Figure 4). Kanthawong et al. found that LL-31, corresponding to the entire helical region in the membrane-bound NMR structure of human LL-37 (Figure 1) [26], is potent against biofilms [51]. A recently reported antibiofilm peptide, SAAP-148, optimized based on a long LL-37 fragment, IG-24 (a C-terminally extended version of GI-20) (reviewed in [15]), is also a long peptide with 24 amino acids [52]. Collectively, these results would suggest that longer LL-37 peptides have antibiofilm advantages.

SK-24 has one more advantage. Our toxicity analysis indicates that, among the peptides derived from the long helix of LL-37 (Figure 1), SK-24, similar to RI-10, is least hemolytic (Figure 7). A possible reason is that the SK-24 peptide has a low hydrophobic content (37%), resembling the parent peptide LL-37 (35%), while GI-20, GF-17, and 17BIPHE2 all have a higher hydrophobic content in the range of 45–47% (Table 1). The poor hemolysis of RI-10 may be attributed to its short sequence length, since its hydrophobic content and hydrophobicity are not lower than those of GF-17, GI-20, and 17BIPHE2. In summary, our structure-based design enabled us to identify a unique peptide, which is potent in microbial killing at low micromolar concentrations, which is unlikely to be toxic to host cells. In addition, SK-24, corresponding to the long helical domain of LL-37 sandwiched between S9 and P32 (Figure 1), appears to contain the minimal sequence for effective oligomerization in PBS. Future high-resolution structural studies of the SK-24 model peptide in PBS may unveil the molecular mechanism of its oligomerization.

## 4. Materials and Methods

### 4.1. Peptides and Chemicals

All peptides were chemically synthesized and purified to >95% (Genemed Synthesis, San Antonio, TX, USA). The quality of each peptide was determined based on mass spectrometry (MS) and high-performance liquid chromatography (HPLC). Peptide stock solutions were freshly made by solubilizing in autoclaved distilled water, and their concentrations were quantitated using UV spectroscopy based on the absorbance difference at 215 and 225 nm as described [18]. Peptide retention time was measured on a Waters C8 symmetry hydrophobic column as described [34]. Other chemicals were purchased from Sigma (St. Louis, MO, USA) unless specified.

### 4.2. Circular Dichroism (CD)

CD spectra were measured on a Jasco J-815 spectropolarimeter in the far-UV region from 260 to 190 nm with a 1 nm interval, a 2 nm bandwidth using a digital integration time of 4 s, and a scan speed of 20 nm per min. During measurement, the high-tension signal applied to the detector was also recorded and was subsequently converted to absorbance. Data represent the average of five individual scans with a corresponding reference measurement on pure solvent subtracted [21,39]. The peptide concentration was fixed at 0.5 mM in 10 mM PBS (pH 7) with or without 60 mM of sodium dodecyl sulfate (SDS). Each sample solution was placed in a 0.1 mm quartz cuvette. The temperature was kept at 25 °C during measurements. Data were processed and converted to molar ellipticity [θ] using the Jasco Spectra Analysis software and plotted using GraphPad Prism 7.

### 4.3. Antibacterial Assays

The antibiotic-resistant pathogens used in this study include the Gram-positive bacteria *Enterococcus faecium* V286-17 and *Staphylococcus aureus* USA300 LAC and the Gram-negative bacteria *Klebsiella pneumoniae* E406-17, *Acinetobacter baumannii* B28-16, *Pseudomonas aeruginosa* E411-17, and *Escherichia coli* E423-17. All bacteria were cultivated in tryptic soy broth (TSB) at 37 °C, 220 rpm.

The antibacterial activity of peptides was evaluated using a standard broth microdilution protocol [53] with minor modifications [40]. In brief, peptides were made at various concentrations, 10 μL per well in a 96-well polystyrene microplate. Logarithmic phase bacterial cultures (i.e., optical density (OD) at 600 nm ≈ 0.5) were diluted to 0.001 OD, and aliquots of 90 μL were added to each well. Untreated bacterial culture and medium were included as positive and negative controls, respectively. Plates were incubated at 37 °C overnight and read at 630 nm using a ChroMate Microplate Reader (Awareness Technology, Palm City, FL, USA). The MIC is the lowest peptide concentration that completely inhibits bacterial growth. Our previously designed peptide 17BIPHE2 [32] was included as a positive control, and RI-10, a peptide without antibacterial activity in rich media [26], was utilized as a negative control.

### 4.4. Antibiofilm Assays

The antibiofilm activities of peptides against the preformed biofilms of *A. baumannii* B28-16 and *S. aureus* USA300 LAC were evaluated as follows. Bacteria in the logarithmic growth phase were adjusted to a cell density of 10^5^ colony forming unit (CFU)/mL. Two hundred microliter cells were seeded into each well of the 96-well plates. The plates were incubated at 37 °C to allow for the formation of biofilm for 24, 48, and 72 h, respectively. The planktonic cells were then removed, followed by a wash. The biofilms were then treated with 20 µL of 10× two-fold diluted peptides and 180 µL of fresh TSB media for 24 h at 37 °C. Cultures treated with water without peptide served as the positive control, and media treated with water was used as the negative control. To quantify the antibiofilm activity of the peptides, the XTT [2, 3-bis (2-methyloxy-4-nitro-5-sulfophenyl)-2*H*-tertazolium-5-carboxanilide] assay staining method was used to quantitate the live cells [54,55,56]. Absorbance was read at 450 nm using a ChroMate microliter plate reader. The percentage biofilm growth for the peptide was plotted by assuming 100% biofilm growth in untreated bacterial control wells. Data were represented as mean ± standard deviation (SD). The data are statistically significant at *p* < 0.05. Plots were generated using GraphPad Prism v7 (GraphPad Software, San Diego, CA, USA).

### 4.5. Membrane Permeabilization

Membrane permeabilization was evaluated as described [39]. In brief, bacteria were inoculated and grown overnight. A second inoculation was made and let bacteria grow to the exponential phase. Bacteria were then spun, washed, and resuspended in the same volume of PBS. A series of peptide solutions (10×) was made via two-fold dilution in a 96-well plate (10 µL each). All wells were aliquoted with 2 µL of propidium iodide (20 µM), followed by adding 88 µL of *S. aureus* USA300 or *A. baumannii* B28-16 (OD_600_ ~0.11 in PBS). The plate was incubated at 37 °C with continuous shaking at 100 rpm in a FLUOstar Omega microplate reader (BMG Labtech Inc., Cary, NC, USA). Fluorescence was recorded every 5 min for 2 h with excitation and emission wavelengths of 485 and 620 nm. The data were analyzed by the vendor’s software using averaged values from duplicated experiments.

### 4.6. Membrane Depolarization of Bacteria

The experiment was conducted as described [39]. In brief, an overnight culture of *S. aureus* USA300 LAC was subcultured in a fresh TSB medium and grown to the exponential phase. Cells were spun using centrifugation and washed twice with PBS, and resuspended in twice the volume of PBS containing 25 mM glucose and incubated at 37 °C for 15 min. For membrane depolarization measurements, 500 nM (final concentration) of the dye DiBAC4 (3) (bis-(1,3- dibutylbarbituric acid) trimethine oxonol) (AnaSpec, San Jose, CA, USA) was added and vortexed gently. Aliquots of 90 μL of the energized bacteria solution were loaded to the wells, and the plate was fed into a FLUOstar Omega microplate reader. Fluorescence was read for 20 min at excitation and emission wavelengths of 485 and 520 nm, respectively, to get dye normalization. Then 10 μL of diluted peptide solutions were added and gently mixed. Fluorescence readings were recorded for 40 min, where Triton X-100 (0.1%) was used as a positive control.

### 4.7. Hemolytic Assays

Human red blood cells (hRBCs, the UNMC Blood Bank) were washed three to five times with saline (Fisher Scientific, Waltham, MA, USA) at 800 g for 10 min each time, until the supernatant was clear [34]. The supernatant was discarded, and hRBCs were suspended in saline at a final density of 2%. Ninety microliters of hRBC suspensions were added to each well of 96-well plates containing 10 µL of 10× serially diluted peptides. After incubation at 37 °C for 1 h, the plates were centrifuged at 1500 rpm for 5 min. About 80 µL of the supernatant was carefully pipetted to a new 96-well microplate and read at 545 nm to measure the amount of hemoglobin released. The positive control was 1% Triton X-100, and the negative control was saline. The percentage of hRBC lysis was calculated based on the hemoglobin released.

## 5. Conclusions

Although there are over 3000 natural antimicrobial peptides in the antimicrobial peptide database, human cathelicidin LL-37 is one of the best characterized antimicrobial peptides discovered to date. Both a library approach and a structure-based design have been utilized to identify active peptides from human cathelicidin LL-37 (reviewed in [15]). Based on the 3D structure of LL-37 [26], we identified the minimal antibacterial peptide KR-12 and minimal antiviral peptide FK-13. In addition, GF-17 is the major antimicrobial and anticancer peptide. Here, we identified SK-24 through a detailed analysis of the 3D-NMR-determined structure of LL-37 bound to membranes (Figure 1). It appears that SK-24 contains the minimal sequence for effective oligomerization of LL-37. Perhaps nature has designed the SK-24 peptide elegantly so that it possesses a stronger antibiofilm ability, but lower toxicity, than its shorter antimicrobial peptides, GI-20 and GF-17, opening the door to potential therapeutic use.

## Figures and Tables

**Table 1:** Peptides, calculated parameters, and measured HPLC retention times.

Peptide ^a^	Net Charge	Pho%	Boman Index	GRAVY	Hydrophobicity	Hydrophobic Moment	t^HPLC^ (min)
LL-37	+6	35	2.99	−0.72	0.201	0.521	12.548
SK-24	+6	37	2.95	−0.69	0.164	0.662	12.291
GI-20	+5	45	2.47	−0.23	0.329	0.729	12.681
GF-17	+5	47	2.47	−0.09	0.378	0.771	12.197
17BIPHE2	+5	47	NA ^b^	NA	NA	NA	10.753
RI-10	+3	50	2.78	−0.01	0.431	0.804	8.831

^a^All LL-37-derived peptides, except for LL-37 itself, are C-terminally amidated. The peptide net charge, hydrophobic content (Pho%), Boman index, and GRAVY were calculated using the antimicrobial peptide database (APD) calculation tool (https://aps.unmc.edu/prediction (accessed on 29 September 2021)) [8]. HeliQuest (Heliquest.ipmc.cnrs.fr) was used to calculate the hydrophobicity and hydrophobic moment [33]. HPLC retention time (t^HPLC^) was measured as described [34]. ^b^ NA, not available.

**Table 2:** Helicity, R1, and R2 calculated from the CD spectra of LL-37 and its fragment peptides in phosphate buffer or bound to membrane mimetic SDS micelles ^a^.

	PBS			60 mM SDS		
Peptide	Helicity	R1	R2	Helicity	R1	R2
LL-37	83.8	−1.94	0.92	97.2	−2.08	0.91
SK-24	105.4	−1.14	0.87	67.6	−2.31	0.86
GI-20	75.1	−2.48	0.98	71.8	−2.40	0.89
GF-17	33.3	−1.91	0.85	54.3	−2.40	0.87
17BIPHE2	7.1	1.18	0.37	25.3	−3.23	1.47
RI-10	9.9	−8.95	24.27	75.5	−2.17	0.78

^a^ R1 is the molar ellipticity ratio of 192/208, while R2 is the ratio of 222/208.

**Table 3:** Bacterial minimal inhibitory concentration (µM) of LL-37 and its derived peptides against drug-resistant pathogens ^a^.

Bacterial Strain	MIC (µM)						
	LL-37	SK-24	GI-20	GI-20d	GF-17	17BIPHE2	RI-10
*E. faecium* V284-17	32	2	2	1	2	2	>32
*S. aureus* USA300	≥32	4	2–4	2	2–4	4	>32
*K. pneumoniae* E406-17	16–32	≥32	>32	>32	>32	4–8	>32
*A. baumannii* B28-16	8	4–8	8	4	4	4–8	>32
*P. aeruginosa* E411-17	>32	>32	>32	32	16	8	>32
*E. coli* E423-17	>32	16	32	32	16	4	>32

^a^Activity evaluated in 100% TSB.

## Data Availability

Data is contained within the article.

## Supplementary Materials

The following are available online at www.mdpi.com/1424-8247/14/12/1245/s1, Figure S1: Effects of LL-37 peptides on the 24 h-formed biofilms of *A. baumannii* and Figure S2: Effects of LL-37 peptides on the 24 h-formed biofilms of *S. aureus*.
