# Supplementary material for: Structure and Activity of a Selective Antibiofilm Peptide SK-24 Derived from the NMR Structure of Human Cathelicidin LL-37
Source: Pharmaceuticals (Basel). 2021 Nov 30;14(12):1245. doi: 10.3390/ph14121245 (PMC8703873; doi:10.3390/ph14121245)

Supporting information

Structure and activity of a selective antibiofilm peptide SK-24 derived from the NMR structure of human cathelicidin LL-37

Yingxia Zhang[‖], Jayaram Lakshmaiah Narayana[‖], Qianhui Wu, Xiangli Dang, and Guangshun Wang[*]

Department of Pathology and Microbiology, College of Medicine, University of Nebraska Medical Center, 985900 Nebraska Medical Center, Omaha, NE 68198-5900, USA

[‖]Major contributors;

*Corresponding author.

Contents

Figure S1. Effects of LL-37 peptides on the 24 h-formed biofilms of *A. baumannii*.

Figure S2. Effects of LL-37 peptides on the 24 h-formed biofilms of *S. aureus*.

Supporting Figures

Figure S1. Antibiofilm activity of (A) LL-37, (B) SK-24, (C) GI-20, (D) GF-17, (E) 17BIPHE2, and (F) RI-10 against *A. baumannii* B28-16. The biofilms were preformed for 24 h.

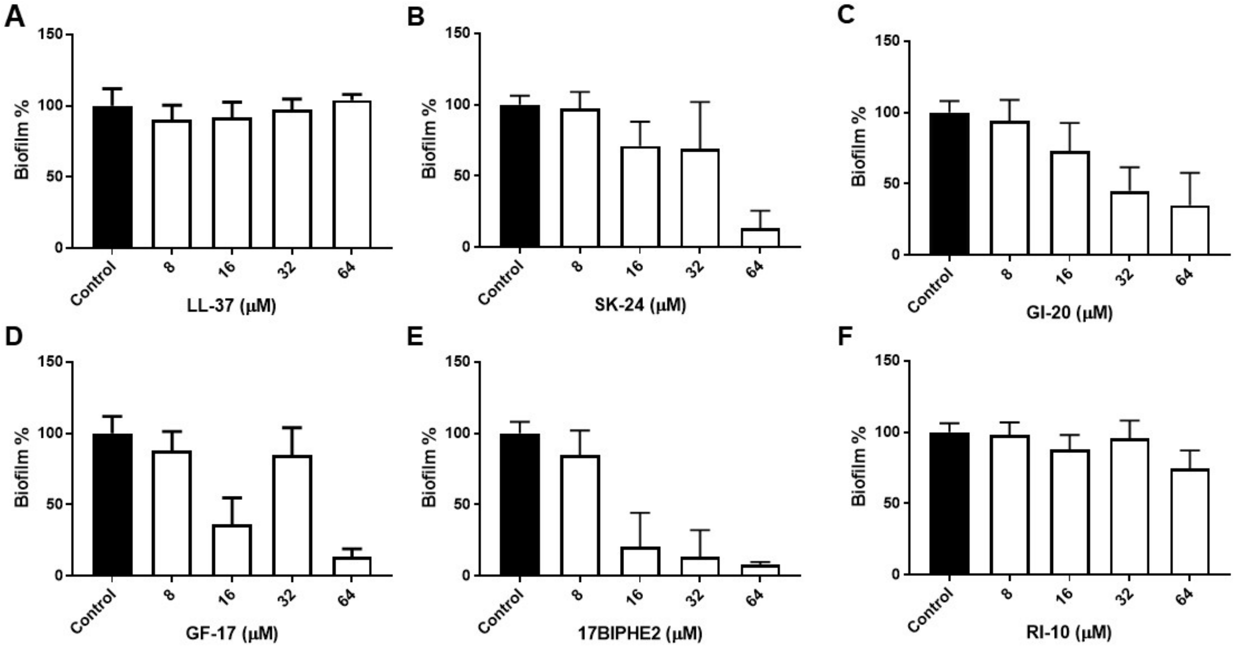

Figure S2. Antibiofilm activity of SK-24, GF-17, GI-20, and 17BIPHE2 against *S. aureus* USA300. Biomasses remained after treatment of the 24-h biofilms were quantitated using crystal violet.

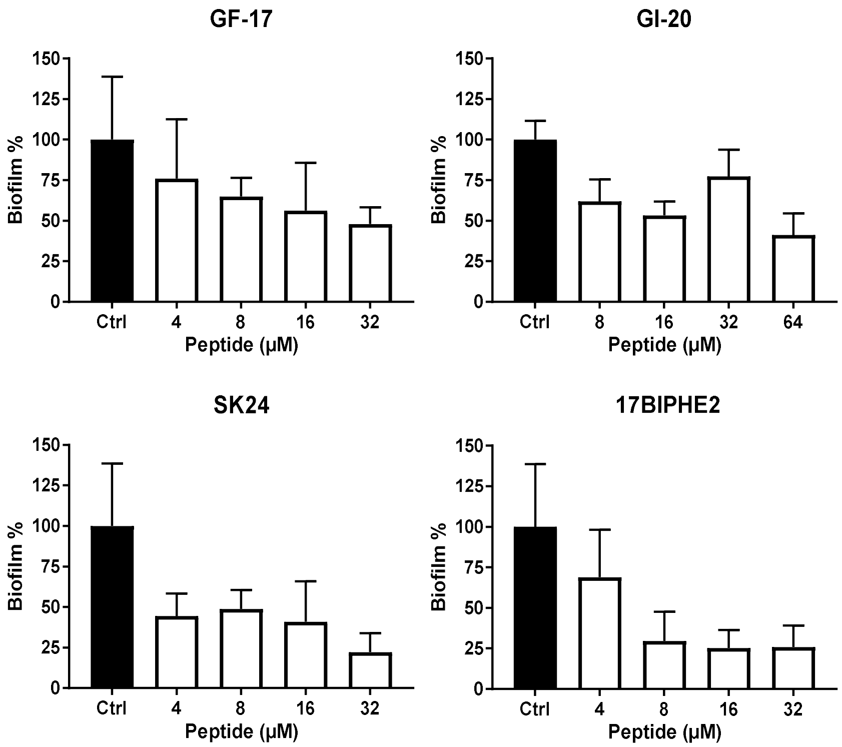

Supplement: Supplementary file 1 [file pharmaceuticals-14-01245-s001.zip › pharmaceuticals-1425343-supplementary.pdf]
